# A Sonographic and Functional Study of the Patterns of Changes in the Tibialis Anterior Muscle in Chronic Hemiplegic Patients, a Pilot Study

**DOI:** 10.3390/biomedicines14081657

**Published:** 2026-07-23

**Authors:** Daniela Poenaru, Claudia Gabriela Potcovaru, Livia Alexandra Ion, Andreea Dumitrescu, Simona Elena Savulescu, Delia Cinteza

**Affiliations:** 1 Rehabilitation Department, Carol Davila University of Medicine and Pharmacy, 020021 Bucharest, Romania; daniela.poenaru@umfcd.ro (D.P.); andreea.petrut@drd.umfcd.ro (A.D.); simona.savulescu@umfcd.ro (S.E.S.); delia.cinteza@umfcd.ro (D.C.); 2 National Institute of Rehabilitation, 030167 Bucharest, Romania; livia-alexandra.ion@rez.umfcd.ro

**Keywords:** stroke rehabilitation, tibialis anterior, musculoskeletal ultrasound, muscle architecture, pennation angle, contraction index, gait velocity, ankle–foot orthosis

## Abstract

**Introduction**: Chronic stroke frequently causes structural and functional impairments in the tibialis anterior (TA) muscle, leading to foot-drop and altered gait kinematics. We investigated the relationship between ultrasonographic parameters of the TA muscle and functional gait performance in chronic stroke survivors. **Methods**: This cross-sectional, observational pilot study evaluated eight consecutive chronic stroke patients (>6 months post-stroke). Structural parameters of TA (rest and contraction thickness, pennation angle) were documented by ultrasound imaging. A dynamic feature calculated was the Contraction Index (CI = effort/resting thickness). Functional metrics included the Medical Research Council (MRC) muscle strength scale and the 10-Meter Walk Test (10MWT) for gait velocity. **Results**: Statistical analysis revealed a moderate negative correlation trend between resting TA muscle thickness and functional gait speed (r = −0.618, *p* = 0.102). Conversely, a moderate positive correlation trend was found (r = 0.548, *p* = 0.160). Ultrasound imaging successfully differentiated three distinct pathological phenotypes: an atrophic phenotype (low pennation angle, flaccid muscle failure), a severely shortened spastic phenotype (increased resting thickness, high pennation angle, pathological CI < 1.0), and a spastic co-contraction loop. Patients with the atrophic phenotype achieved high mechanical efficiency with an ankle–foot orthosis (AFO), whereas the spastic phenotype exhibited resistance against the orthotic device. **Conclusions**: Musculoskeletal ultrasound provides objective parameters for post-stroke TA muscle remodeling and contributes to completing the assessment and therapy. Identifying specific structural muscle phenotypes allows clinicians to optimize target-specific neurorehabilitation strategies, predict AFO efficiency, and guide antispastic interventions such as botulinum toxin injections.

## 1. Introduction

Stroke remains a leading cause of mortality and long-term adult disability worldwide, imposing a substantial clinical and socioeconomic burden on global healthcare systems [1]. While acute medical interventions have significantly improved survival rates, a vast majority of stroke survivors face permanent functional impairments. Among these sequelae, chronic gait and mobility disability is highly prevalent, severely compromising patient autonomy and quality of life. Gait rehabilitation is a main objective of all training programs. Lower limb complex evaluation is followed by targeted therapies to address spasticity, weakness, muscle imbalance and pathological movements [2].

The tibialis anterior (TA) muscle is anatomically located within the anterior compartment of the lower leg, originating from the proximal lateral surface of the tibia and inserting via its distal tendon into the medial aspect of the first cuneiform and the base of the first metatarsal [3].

Functionally, the TA serves as the primary dorsiflexor of the foot and, acting synergistically with the tibialis posterior, executes foot inversion. The TA exhibits critical biomechanical activity across specific phases of the gait cycle. At initial contact (heel strike), it undergoes eccentric contraction to decelerate plantarflexion, thereby preventing foot-drop. Conversely, following toe-off and throughout the swing phase, the muscle contracts concentrically to achieve sufficient dorsiflexion for foot clearance.

Chronic stroke significantly disrupts the neuromuscular activation and recruitment patterns of the tibialis anterior. While isolated spasticity of the TA is an uncharacteristic clinical presentation, its co-activation with a spastic tibialis posterior frequently manifests as a fixed varus foot deformity. More commonly, post-stroke upper motor neuron syndrome features predominant spasticity within the triceps surae complex. This imbalance culminates in an equinovarus foot posture, resulting in a clinical drop-foot that necessitates maladaptive compensatory kinematic strategies to ensure adequate foot clearance during locomotion [4].

Consequently, the tibialis anterior (TA) muscle serves as a primary therapeutic target for post-stroke motor rehabilitation strategies. Functional electrical stimulation (FES), applied directly over the muscle belly, is widely utilized to enhance gait speed and restore lower limb symmetry [5,6]. Similarly, neurorehabilitation modalities such as electromyographic (EMG) biofeedback, neuromuscular electrical stimulation (NMES), and kinesio taping have been extensively documented to target identical functional outcomes. Ankle–foot orthosis (AFO), in many different designs, is prescribed as a compensatory intervention to maintain proper ankle–foot alignment, prevent foot drop, reduce tone, and stabilize the ankle during locomotion.

While post-stroke gait impairment is primarily driven by central nervous system command failure and disrupted neuromuscular coordination, the chronic absence of physiological recruitment triggers severe, secondary peripheral remodeling within the muscle belly. Therefore, we focused on evaluating these peripheral adaptations in the tibialis anterior muscle to map how chronic neurological deficits translate into distinct structural tissue phenotypes.

## 2. Materials and Methods

This study was designed as an observational, descriptive, and analytical cross-sectional study focusing on chronic stroke survivors.

Participants were consecutively recruited from the pool of patients admitted to the Rehabilitation Department. To be eligible for inclusion, patients had to meet the following criteria: (1) a documented history of chronic stroke with a time lapse of more than 6 months since the acute event, (2) the ability to ambulate either independently or with the assistance of mobility devices, and (3) the cognitive and communicative capacity to cooperate and interact effectively with the rehabilitation team.

Exclusion criteria were: (1) active or historical lumbar spinal disease (e.g., radiculopathy or spinal stenosis) that could interfere with lower limb motor output, or (2) a history of traumatic events or orthopedic surgeries involving spine and/or the lower legs.

Demographic and baseline clinical characteristics were recorded for all participants, including sex, age, stroke localization (ischemic/hemorrhagic and hemispheric side), and the exact time lapse since the stroke onset (measured in months).

Motor and functional parameters were evaluated using two validated clinical tools:

1. Muscle Strength: Volitional strength of the tibialis anterior (TA) muscle was graded using the Medical Research Council (MRC) scale, a 6-point system ranging from 0 (no muscle contraction) to 5 (normal muscle strength against full resistance).
2. Gait Velocity: The 10-Meter Walk Test (10MWT) was employed to evaluate walking speed. Patients were instructed to walk at their comfortable, habitual pace over a total distance of 10 m. Three consecutive determinations were averaged for a final value. The use of daily assistive devices (such as canes, crutches, or ankle–foot orthoses) was permitted and documented.

Structural parameters of the tibialis anterior muscle were assessed using a diagnostic ultrasound machine equipped with a high-resolution linear probe (8–14 MHz, ARIETTA 650 DeepInsight Fujifilm Healthcare Corporation, Tokyo, Japan) operating in grayscale (B-mode). For all sonographic evaluations, patients were positioned in a standardized, relaxed supine posture on an examination table, with both knees extended and the ankle joints positioned in a neutral resting angle (~90° flexion), supported by a customized foam wedge to eliminate gravity assistance. To record active contraction parameters, patients were given standardized verbal encouragement (‘pull your foot up towards your shin as hard as possible’) and performed three separate maximal voluntary isometric dorsiflexion contractions against stable manual resistance provided by the examining physician, holding each effort for 5 s. To maximize intrarater reliability and control for measurement error, all sonographic evaluations were executed by a single expert sonographer with over 20 years of musculoskeletal experience. Reliability was operationally optimized by enforcing a strict non-compression probe technique and recording the arithmetic mean of three distinct, consecutive acquisitions for every architectural metric.

The following parameters were recorded:

Muscle thickness was defined as the maximum distance between the superficial fascia and the deep fascial interface of the muscle belly. For each patient, three consecutive measurements were obtained and averaged to record: (1) TA muscle thickness on the affected side at rest, (2) TA muscle thickness on the affected side during maximal voluntary isometric contraction (dorsiflexion), and (3) TA muscle thickness on the unaffected side at rest.

The pennation angle, defined as the angle between the deep fascial sheath and the muscle fascicles, was measured at the same standardized 10 cm infra-patellar landmark on both the affected and unaffected limbs, and three consecutive measurements were performed to obtain an average value.

## 3. Results

Statistical Analysis: Statistical analysis was performed using the SPSS software package (IBM SPSS Statistics for Windows, Version 26.0; IBM Corp., Armonk, NY, USA).

This preliminary investigation evaluated the first eight consecutive patients as a pilot study to document baseline structural and functional evolutions. Continuous variables (age, time elapsed since stroke, muscle thickness, pennation angle, and gait speed) were initially tested for normality using the Shapiro–Wilk test. Given the small sample size (*n* = 8) and the normal distribution of the primary metrics, descriptive statistics were expressed as means ± standard deviations (SD), medians, and operational ranges (minimum to maximum values). Categorical data (sex, affected side, and assistive device type) were presented as absolute frequencies and percentages. To determine the strength and direction of the linear relationship between resting tibialis anterior muscle thickness and functional gait speed, a Pearson correlation coefficient (r) was computed. The level of statistical significance was strictly set a priori at *p* < 0.05.

### 3.1. Demographic Analysis of the Pilot Group Is Displayed in the Following Table 1

To evaluate the functional dynamics of the tibialis anterior muscle during volitional activity, we used a dynamic parameter, the Contraction Index (CI), calculated for each patient by computing the ratio of muscle thickness during maximal voluntary contraction to muscle thickness at rest. CI (Effort Thickness/Rest Thickness) expresses the functional performance of the selected muscle.

**Table 1:** Demographic and clinical baseline characteristics of the chronic stroke survivors (*n* = 8). Continuous variables (age and time elapsed since stroke) are expressed as mean ± standard deviation, median, and range. Categorical variables (sex and affected side) are expressed as absolute frequencies and percentages.

Parameter	Patient-Specific Data/Cohort Metrics
Sex, (*n*) (%)	
—Male	5 (62.5%)
—Female	3 (37.5%)
Affected Side, (*n*) (%)	
—Right (DR)	4 (50.0%)
—Left (STG)	4 (50.0%)
Age (years)	
—Mean ± SD	54.62 ± 14.31
—Median	56.00
—Range (Min–Max)	35.00–76.00
Time elapsed since stroke (months)	
—Mean ± SD	40.62 ± 27.71
—Median	43.50
—Range (Min–Max)	7.00–75.00

Structural parameters, including bilaterally assessed resting thickness, active contraction thickness on the affected side, and corresponding pennation angles, were noted for each patient and individual functional parameters such as Medical Research Council (MRC) strength scores and 10-Meter Walk Test (10MWT) gait velocities. They are detailed in Table 2.

The relationship between tibialis anterior muscle thickness at rest and gait speed was investigated.

### 3.2. Relation Between Tibialis Anterior Muscle Thickness at Rest and Gait Speed Is Displayed in Figure 1

A Pearson correlation coefficient was calculated to evaluate the linear relationship between resting TA muscle thickness (cm) and functional gait speed (m/s) across the pilot cohort. The statistical analysis revealed a moderate negative correlation trend between the two primary variables (r = −0.618, r^2^ = 0.381, *p* = 0.102). Although the relationship did not cross the strict threshold for statistical significance due to the limited sample size of this pilot study, the high negative correlation coefficient (r) corroborates a clear clinical trend: a higher resting TA muscle thickness is robustly associated with a lower, more restricted walking speed.

**Figure 1:**
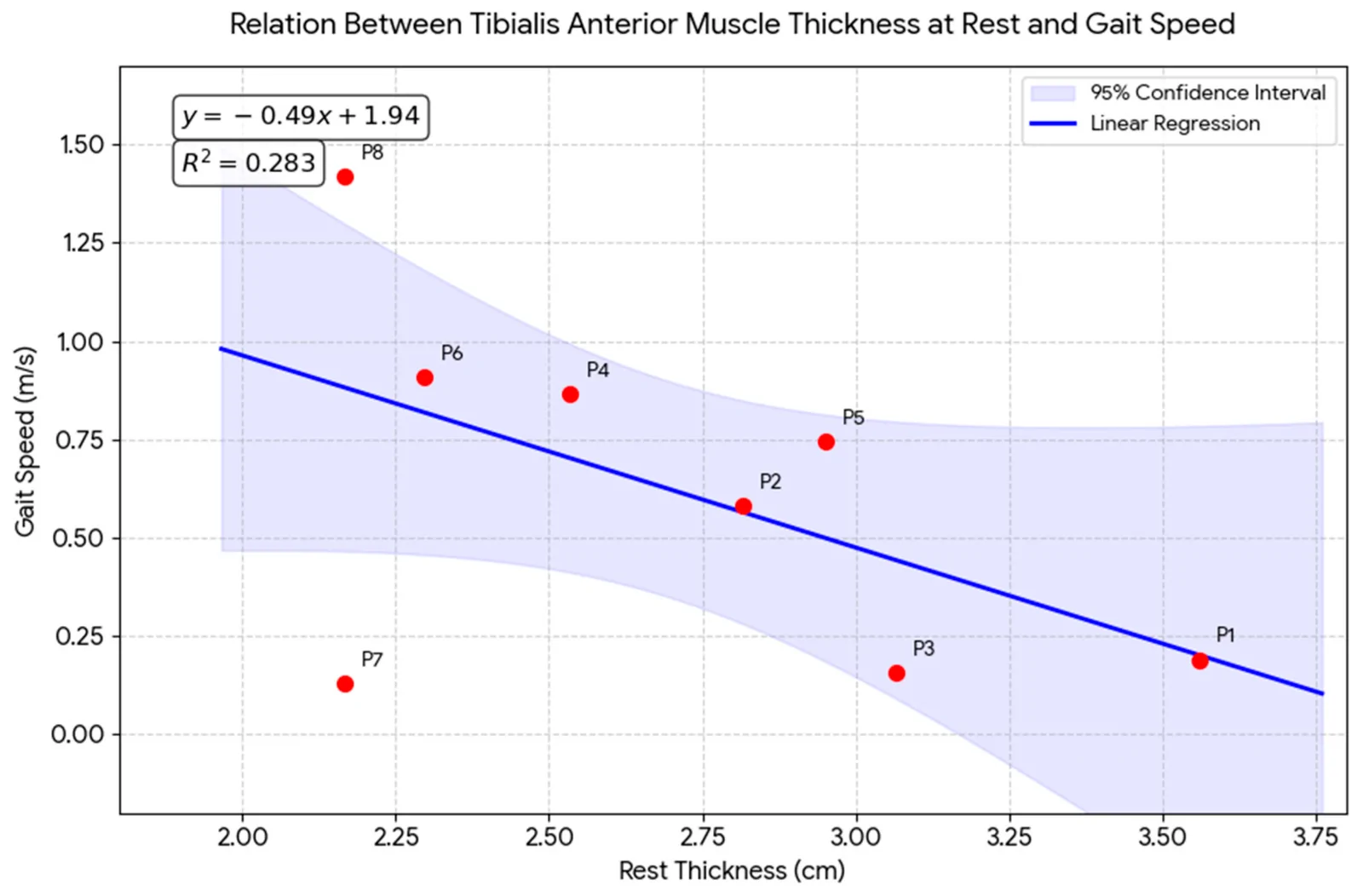
Scatter plot representing the negative linear relationship between resting TA muscle thickness (cm) and functional gait speed (m/s) in chronic stroke survivors. The downward slope (r = −0.618) demonstrates how a thicker muscle profile at rest consistently tracks with slower walking speeds due to spastic structural remodeling and non-functional tissue bulk.

### 3.3. Relation Between CI and Gait Speed Is Displayed in Figure 2

A Pearson correlation coefficient was calculated to analyze the relationship between the functional performance (expressed by CI) of the TA muscle and functional gait speed (m/s) across the cohort (*n* = 8). The analysis revealed a moderate positive correlation trend between the two variables (r = 0.548, r^2^ = 0.300, *p* = 0.160). Although the clinical trend did not cross the strict threshold for statistical significance due to the limited sample size of this pilot study, the positive correlation coefficient (r) highlights a clear functional trajectory: a higher capacity of the muscle to contract and thicken actively corresponds to an increased and more functional walking speed.

We made some preliminary observations regarding the studied patients.

While the small sample size limits statistical significance, the clinical data highlights a striking exception: Patient P1 demonstrated a pathological CI below 1.0 (CI = 0.924), indicating an absolute loss of active muscular recruitment, which directly corresponds to severe gait impairment (0.190 m/s).

**Figure 2:**
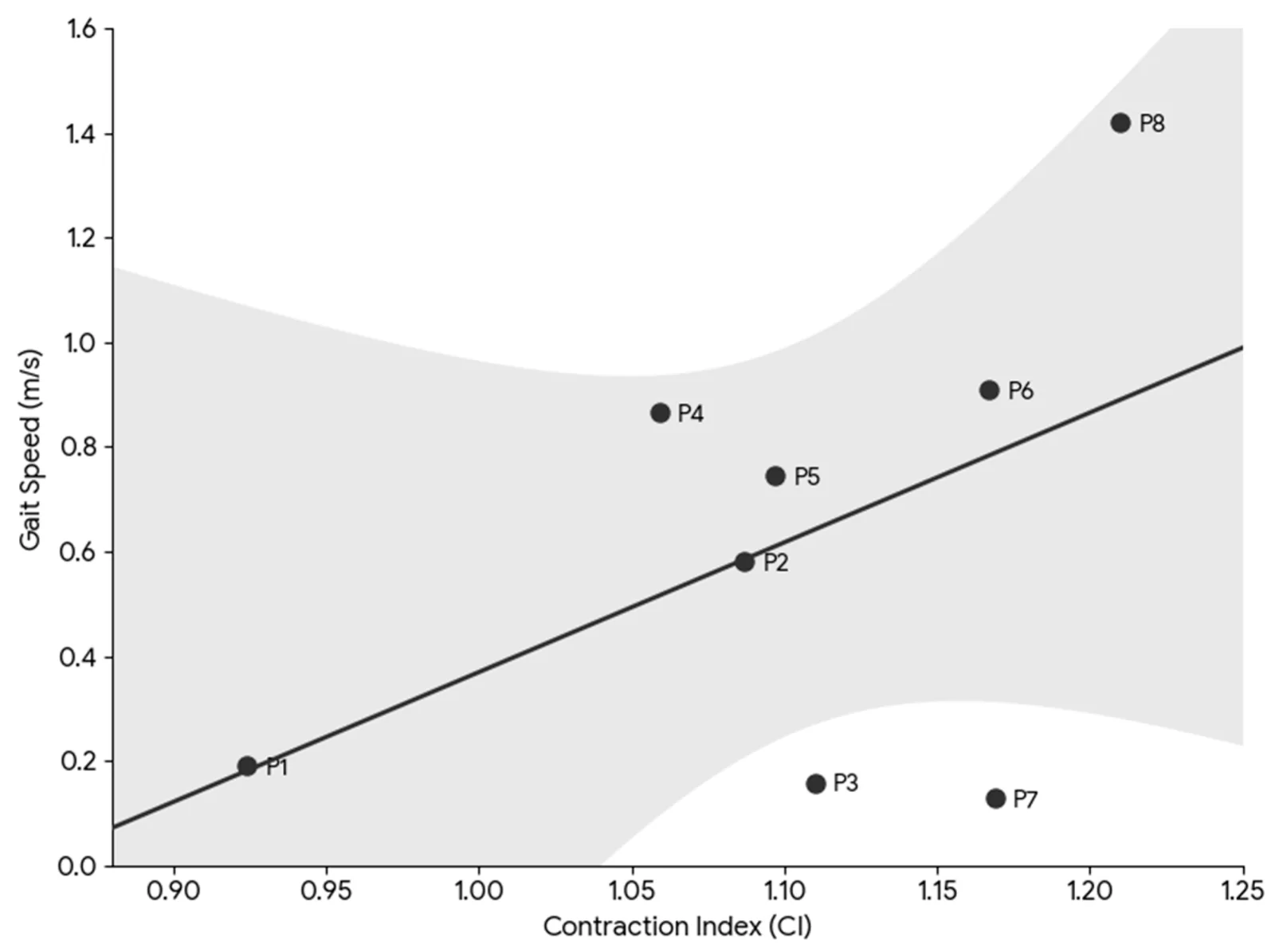
Scatter plot depicting the positive linear relationship between the dynamic CI and gait velocity (m/s) in chronic stroke survivors. The upward regression line (r = 0.548) indicates that preserved neuromuscular recruitment and active muscle thickening directly track with enhanced gait capacity. Patient 1 (P1) drops strictly below the critical 1.0 threshold line, capturing a paradoxical muscle thinning failure. The grey shaded area indicates the region were the CI is above the 1.0 treshold, representing structural muscle thickening during gait.

Patient P3 presents a clinical paradox that underlines the limitations of assessing muscle function through ultrasound in isolation. This patient demonstrated a calculated CI above 1.0 (CI = 1.101, structural thickening from 3.066 cm to 3.376 cm), which would typically suggest preserved muscle recruitment. However, this architectural thickening was paradoxically associated with the lowest functional outcome in the entire cohort: a severely impaired gait speed of just 0.158 m/s. In a healthy subject, a 10.1% increase in muscle thickness during effort signifies an effective concentric contraction that generates ankle dorsiflexion. In the context of chronic stroke sequelae, however, P3’s ultrasound pattern reflects an entirely different neuro-biomechanical phenomenon:

1. Spastic co-contraction and ankle locking: The thickening documented on ultrasound during contraction is not a result of isolated, voluntary motor unit recruitment of the TA. Instead, the voluntary command triggers a pathological, undifferentiated mass synergy. This causes a simultaneous, massive co-contraction of both the tibialis anterior (agonist) and the highly spastic triceps surae complex (antagonists/calf muscles). The muscle bulks up because it is locked in a rigid isometric contraction, which stabilizes the joint but completely prevents the dynamic ankle excursion needed to clear the foot during the swing phase of walking.
2. Fibro-elastic changes and false hypertrophy: P3 also displays a high resting thickness (3.066 cm), which ranks among the highest in the study. This structural bulk, combined with the inability to translate contraction into movement, indicates that the muscle tissue has undergone significant remodeling (fibrosis and fat infiltration). The tissue is stiff and loses its compliance, requiring massive, non-functional neurological effort just to alter its shape, without producing any mechanical work.

Therefore, P3’s data delivers an important conclusion for the study: a positive CI post-stroke does not guarantee functional gait unless it is accompanied by selective, coordinated motor control free from antagonist spastic interference.

Patient P6 had the highest CI (1167), a physiological CI above 1.15.

### 3.4. Architectural Muscle Analysis via Pennation Angle Is Pictured in Figure 3

Ultrasonographic architectural assessment revealed substantial, highly divergent patterns of structural adaptation in the TA muscle across the cohort. The pennation angle provided direct morphological evidence of chronic neuromuscular remodeling, mapping tightly with individual spastic and atrophic clinical phenotypes.

**Figure 3:**
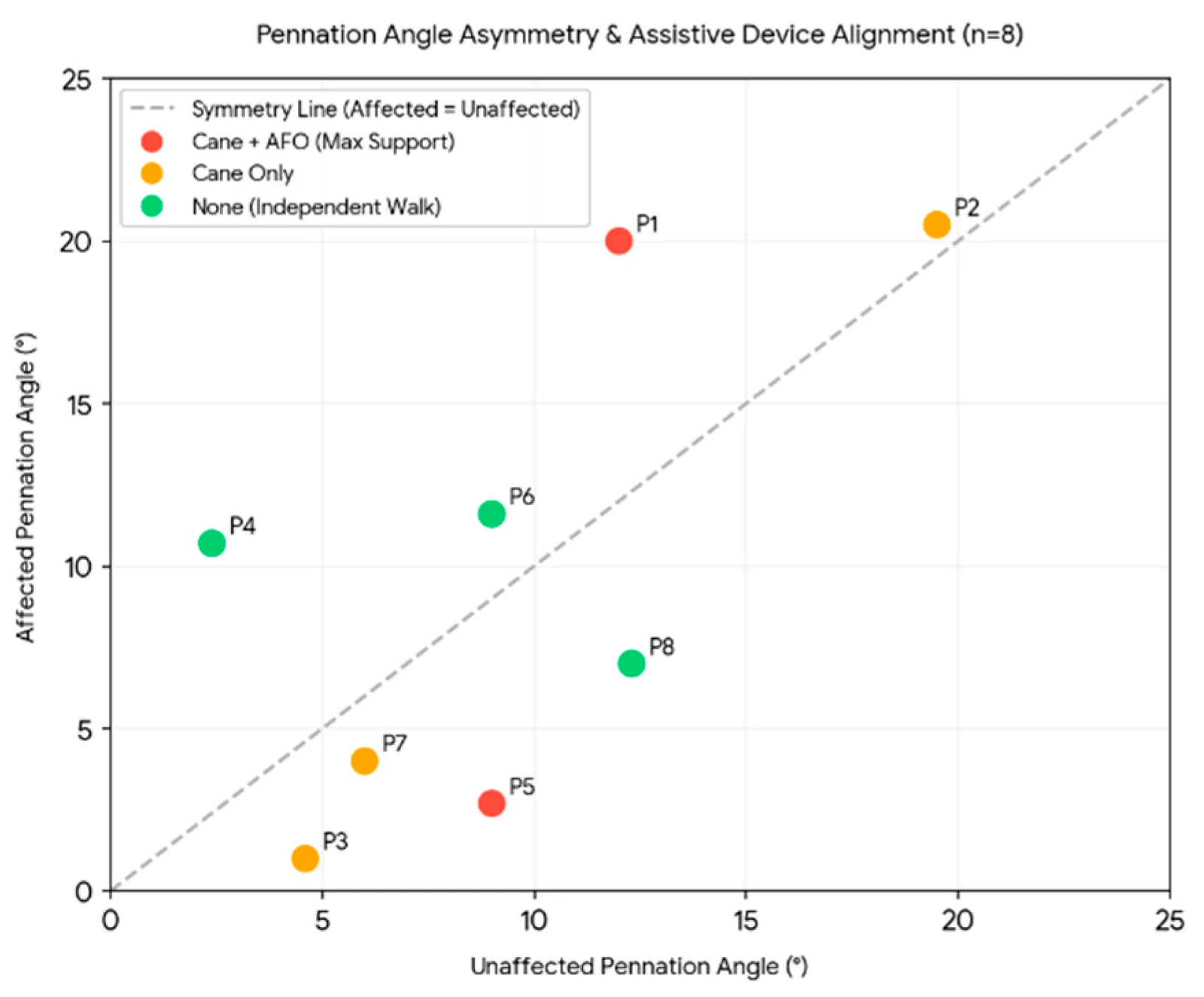
Scatter plot of the TA pennation angle asymmetry, color-coded by mobility assistive device dependency. Points sitting directly on or close to the dashed diagonal symmetry line (such as P2, P6, and P4) indicate preserved or functional muscle architecture. Deviations far above the line (P1) indicate spastic shortening and vertical fascicle realignment, while drops far below the line (P3, P5, P7, and P8) represent severe atrophic collapse and parallel muscle fiber deletion.

Among the chronic stroke survivors, a clear architectural distribution emerged based on the predominant upper motor neuron syndrome presentation:

1. Severely shortened spastic architecture (P1): Patient P1, presenting with marked focal spasticity and fixed plantarflexion resistance, demonstrated an abnormally increased pennation angle on the affected side compared to the unaffected limb (20.00° vs. 12.00°). This structural realignment indicates a severe spatial compaction of the muscle belly. Chronic hypertonia forces the fascicles into a highly vertical orientation, which actively increases resting thickness while severely limiting the dynamic range of motion.
2. Preserved architectural symmetry with moderate function (P2): In contrast, Patient P2 exhibited a remarkably well-preserved architectural symmetry between limbs, presenting only a minor, non-pathological asymmetry of 1.00° (20.50° on the affected side vs. 19.50° on the unaffected side). This high degree of structural preservation strongly correlates with the patient’s residual functional capacity, as evidenced by a preserved volitional strength score of MRC 3+ and a functional, independent gait speed of 0.580 m/s using only a cane for external balance.
3. Atrophic/collapsed architecture (P3, P5, and P7): Conversely, a severe architectural collapse of the pennation angle was documented in patients characterized by advanced disuse atrophy, flaccid paresis, or mass synergistic failure. Patient 3 and Patient 5 exhibited minimal operational thresholds on the affected side (1.00° and 2.66°, respectively). This flat structural alignment represents a profound loss of parallel muscle fascicle volume and contractile mass. Similarly, Patient 7, despite a low resting thickness (2.167 cm) and a flat pennation angle (4.00°), was restricted to a severely impaired walking velocity (0.129 m/s, using a cane), highlighting how severe architectural collapse and muscle wasting compromise foot clearance during locomotion.
4. Optimal architectural symmetry (P4, P6, and P8): Independent community ambulators who walked without assistive devices displayed highly symmetric or optimal architectural configurations. Patient 8 achieved the highest velocity in the study (1.420 m/s) while exhibiting a well-preserved, flexible pennation angle of 7.00° on the paretic side, further confirming that the absence of severe architectural distortion is a prerequisite for generating coordinated concentric work.

Consequently, evaluating pennation angle asymmetry via musculoskeletal ultrasound serves as an objective marker for tissue viability post-stroke. Discriminating between a rigid spastic architecture (high asymmetry), an atrophic/collapsed profile (severe angle drop), and a well-preserved symmetric baseline (as seen in P2) provides crucial diagnostic insight, directly influencing clinical decisions regarding targeted rehabilitation protocols.

### 3.5. Correlation of Ultrasonographic Parameters with Assistive Device Dependency Is Depicted in Figure 4

The clinical requirement for mobility assistive devices in this cohort demonstrated a strong vertical alignment with the structural and architectural degeneration of TA. By grouping patients based on their level of ambulatory assistance, musculoskeletal ultrasound metrics exposed the underlying morphological changes dictating functional dependency:

1. The independent ambulation level (None—P4, P6, P8):

Patients who successfully ambulated without any external support achieved optimal, high-velocity community gait patterns (>0.85 m/s, up to 1.420 m/s in P8). Ultrasonographically, this sub-group was defined by the preservation of physiological muscle architecture. Their resting muscle thickness remained low and compliant (ranging from 2.166 cm to 2.533 cm), they displayed correct active thickening capacities (yielding the study’s highest CI, up to 1.210), and their pennation angles remained symmetric or flexible. This indicates conservation of normal architecture and normal elastic properties (an absence of dense tissue fibrosis, permanent sarcomere shortening, or severe muscle wasting). TA offers the necessary mechanical compliance for efficient ankle excursion during locomotion.

2. The moderate assistance level (Cane only—P2, P3, P7):

Patients relying solely on a cane for external balance displayed highly heterogeneous structural profiles, revealing distinct pathophysiological coping mechanisms. Patient 2 maintained near-perfect architectural symmetry (pennation angle asymmetry of only 1.00°), allowing for a preserved volitional strength of MRC 3+ and a functional gait speed of 0.580 m/s. Conversely, P3 and P7 presented with advanced muscle degradation but diverged in neuromuscular control. P7 exhibited an atrophic/collapsed architecture (affected pennation angle of 4.00° and MRC 1) that restricted walking speed to a severely impaired 0.129 m/s. Patient 3 displayed a false structural hypertrophy at rest (3.066 cm) and an isometric thickening during effort (CI = 1.101), but achieved a very low speed of 0.158 m/s. This indicates a rigid spastic co-contraction loop where both agonists and antagonists contract simultaneously, converting the muscle bulk into rigid joint stiffness rather than functional movement. In this patient, the cane primarily compensates for a loss of forward propulsive power or unilateral balance.

3. The maximum support level (Cane + AFO—P1, P5):

A biomechanical paradox was revealed within the sub-group dependent on an AFO coupled with a cane. While both patients demonstrated advanced TA muscle failure on ultrasound, their functional velocities diverged drastically: P5 achieved an excellent functional speed of 0.750 m/s, whereas P1 was severely limited to 0.190 m/s.

**Figure 4:**
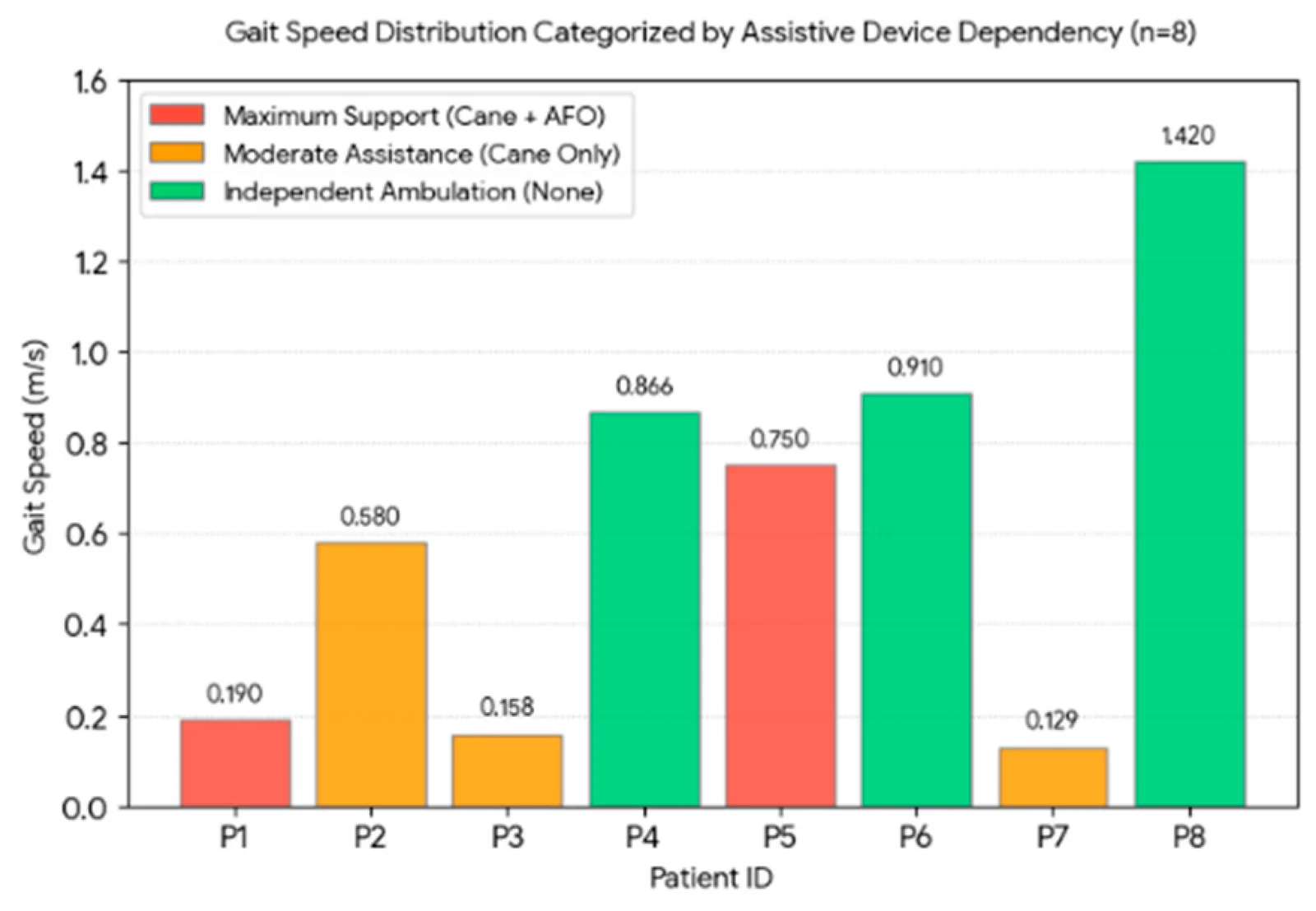
Bar chart illustrating individual functional gait velocities (m/s) across the cohort, color-coded by the level of mobility assistive device dependency. The graphic clearly captures the heterogeneous functional outcomes within identical interventions, most notably the mechanical efficiency paradox between Patient 5 (0.750 m/s) and Patient 1 (0.190 m/s) under identical orthotic and balance support (Cane + AFO).

**Figure 5:**
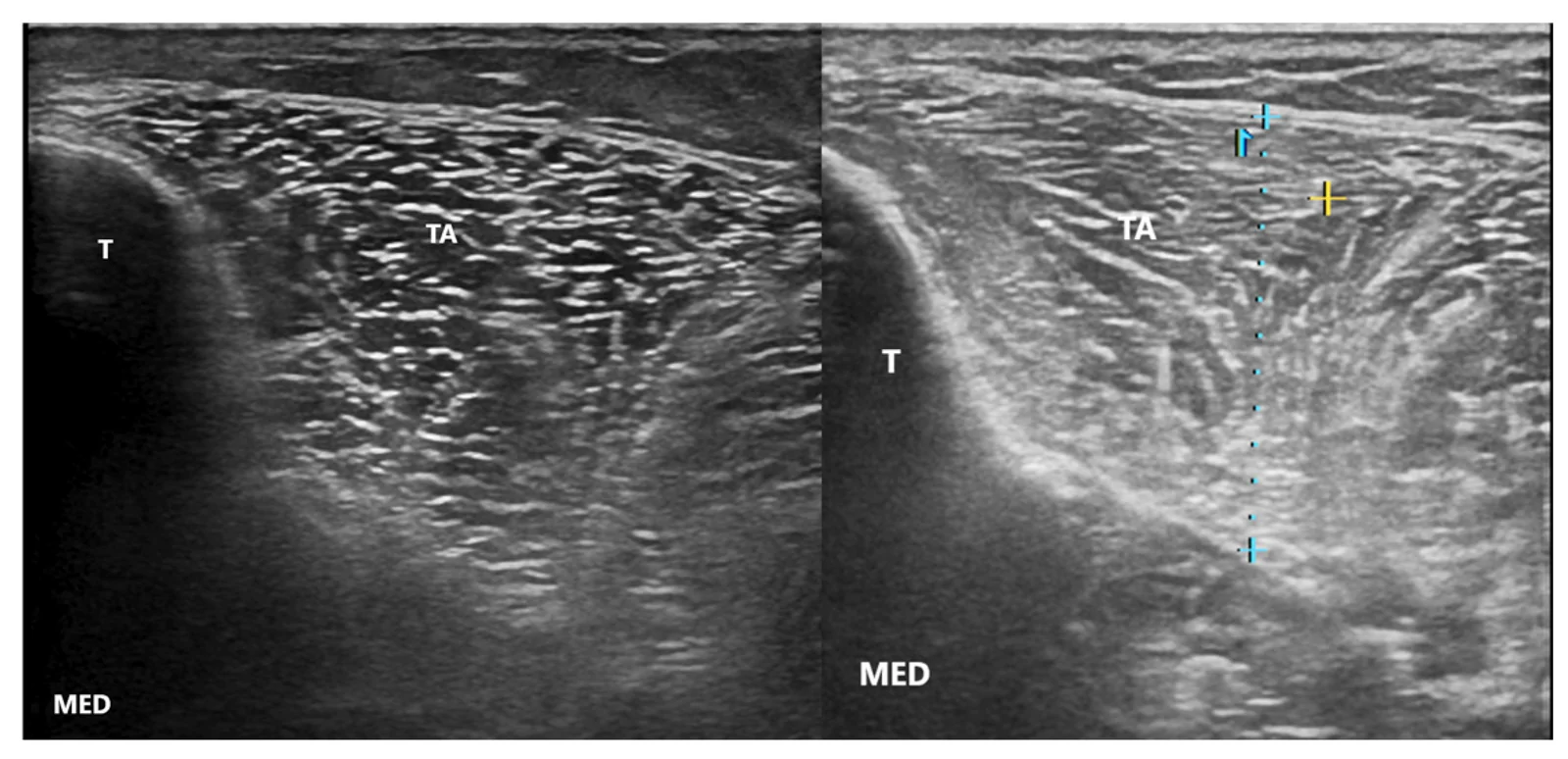
Short-axis section on tibialis anterior (TA) muscle at a precise landmark situated 10 cm distal to the apex of patella, in high-resolution B-mode ultrasound, that illustrates two distinct architectural profiles of our highlighted structural phenotypes (the atrophic phenotype—**left** vs. the compacted spastic phenotype—**right**). T, Tibia; TA, tibialis anterior muscle; yellow dotted line, the calipers position to measure muscle thickness.

Ultrasound parameters resolved this clinical mismatch, proving that AFO mechanical efficiency is heavily modulated by the specific structural phenotype of the paretic muscle (see Figure 5, left, the atrophic aspect: right, the spastic aspect):

- The atrophic AFO pattern (P5): Ultrasound revealed advanced muscle wasting and an atrophic collapse of the pennation angle to 2.66°. Because the muscle is flaccid and offers no active mechanical resistance, the AFO functions as a perfect passive substitute. It neutralizes foot-drop and ensures safe toe clearance, allowing the patient to effectively maximize proximal hip and knee power to maintain a rapid gait velocity.
- The spastic AFO pattern (P1): P1 exhibited advanced structural shortening, characterized by a massive resting bulk (3.560 cm), an abnormally increased pennation angle (20.00°), and a pathological CI below 1.0 (0.924). This structural profile indicates dense fibrosis and permanent hypertonia. Here, the active spastic muscle bulk actively fights against the rigid brace. The AFO ceases to be a simple passive substitute and instead becomes an opposing mechanical force against dense spastic resistance, drastically increasing the energy cost of walking and restricting gait to a non-functional speed.

Consequently, our findings suggest that the prescription and the clinical success of an AFO in chronic stroke rehabilitation are based on a critical threshold of TA architectural failure. Musculoskeletal ultrasound is able to differentiate between flaccid muscle failure (where bracing is highly effective) and structurally remodeled spastic hypertonia (where orthotic interventions must be preceded by targeted antispastic treatments, such as botulinum toxin injections).

## 4. Discussion

Gait after stroke is one of the most important objectives and is part of the independent life of the survivors. The TA muscle, with its delicate eccentric and concentric contraction, ensures the stability of the ankle and an appropriate amount of foot clearing. Furthermore, TA, as a superficial and frequently affected structure, is subject to various research to document cortical and corticospinal pathways in stroke survivors [7].

The findings of this cross-sectional observational pilot study evaluate the complex relationship between TA muscle architecture and functional locomotion in chronic stroke survivors. By utilizing musculoskeletal ultrasound, this study investigates beyond traditional, subjective clinical scales to characterize the structural remodeling of the TA muscle.

A prominent finding of our quantitative analysis is the moderate negative correlation trend between resting TA muscle thickness and functional gait velocity (r = −0.618, *p* = 0.102). While in healthy populations an increased cross-sectional area or muscle thickness directly corresponds to superior force generation and walking speed, our data demonstrates that this physiological rule is fundamentally inverted in chronic stroke patients. A thicker TA muscle profile at rest consistently tracked with lower ambulatory velocities (as seen in P1 and P3). Clinically, this structural–functional mismatch indicates that increased muscle thickness in a paretic limb does not represent functional hypertrophy. Instead, it reflects a pathological combination of chronic spastic shortening, continuous involuntary background recruitment (spastic dystonia), and the replacement of contractile myofibers with non-functional fibro-adipose tissue. Conversely, patients who maintained a thinner, more physiological muscle mass at rest (such as P6 and P8) demonstrated excellent community walking speeds exceeding 0.90 m/s, suggesting a preservation of physiological tissue elasticity and a lower degree of structural degeneration.

It must be underlined that gait velocity is a global parameter, influenced by the structural state of the triceps surae, quadriceps, and hip stabilizers, rather than the TA muscle in isolation. The correlation trends identified in our pilot cohort (r = −0.618 and r = 0.548 do not imply that TA architecture solely dictates walking speed. Instead, they demonstrate how severe structural remodeling of a critical focal link—the primary foot dorsiflexor—can alter the entire lower limb kinetic chain by causing mechanical resistance or flaccid drop-foot, thereby restricting overall velocity.

This architectural differentiation is further underlined by the functional parameter (CI) and the pennation angle. Under normal physiological conditions, the CI remains comfortably above 1.0, as muscle fibers shorten and widen during active volitional recruitment. This healthy pattern was well-preserved in our high-performing independent ambulators (e.g., P8: CI = 1.210, Gait Speed = 1.420 m/s; P6: CI = 1.167, Gait Speed = 0.910 m/s).

In patients with severe neurological deficits, ultrasound imaging exposed three entirely distinct pathological phenotypes that modulate gait outcomes and assistive device dependency:

1. The atrophic phenotype (The P5 and P7 Pattern): Patient 5 and Patient 7 presented with advanced muscular atrophy and a severely collapsed pennation angle on the affected side (2.66° and 4.00°, respectively), representing a profound loss of parallel muscle fascicle volume and contractile mass. However, their functional coping pathways diverged sharply. Patient 5 achieved a remarkably high walking speed of 0.750 m/s due to the mechanical efficiency of the prescribed AFO. Because the atrophic muscle is flaccid and offers no active mechanical resistance, the AFO functions as a perfect passive substitute, neutralizing foot-drop, ensuring safe toe clearance during the swing phase, and allowing the patient to fully leverage proximal hip and knee power. Conversely, Patient 7 lacked this orthotic optimization, resulting in a severely restricted velocity of 0.129 m/s with a cane alone, demonstrating how severe architectural collapse compromises gait when uncompensated. The necessity of an additional AFO is evident in P7.
2. The severely shortened spastic phenotype (The P1 Pattern): Patient 1 demonstrated a completely contrasting adaptive pathway, exhibiting severe structural shortening marked by a massive resting bulk (3.560 cm) and an abnormally increased pennation angle (20.00°). Furthermore, P1 displayed a pathological CI below the functional baseline (CI = 0.924), meaning the muscle paradoxically thinned during attempted contraction. This signifies an absolute failure of volitional motor unit recruitment; when dorsiflexion is attempted, the paretic TA is mechanically overpowered and passively stretched by the hyper-reflexic, spastic triceps surae complex (antagonist). The possible mechanism of triceps surae activation during dorsiflexion command is the irradiation of the excitation at the spinal level lacking inhibitory descending impulses. Although P1 and P5 wore an identical assistive combination (Cane + AFO), their walking speeds differ significantly (0.190 m/s for P1 vs. 0.750 m/s for P5). This proves that the therapeutic utility of an AFO is severely compromised when it must constantly fight against intense hypertonic antagonist, hence the utility of antagonist botulinum administration.
3. Spastic co-contraction (The P3 Pattern): Patient P3 presented an adequate CI (1.101) but the second-lowest walking speed in the study (0.158 m/s). In P3, this architectural thickening does not represent useful concentric contraction, but rather a rigid co-contraction loop where both agonists and antagonists contract simultaneously. Due to differences in the power and the volume of the agonists (TA) and antagonists (triceps surae muscle mainly), the ankle is locked in plantar flexion. Hence, botulinum toxin administration to the antagonists and AFO prescription are necessary, together with evaluation of proximal and intermediate lower limb pivots.

In contrast to these damaged profiles, Patient 2 exhibited a remarkably well-preserved architectural symmetry between limbs (pennation angle asymmetry of only 1.00°), which strongly correlates with a preserved volitional strength of MRC 3+ and a functional walking speed of 0.580 m/s using only a cane for external balance.

Age demographics represent another critical variable when interpreting muscle structural changes. The mean age of our cohort was approximately 55 years, reflecting a middle-aged post-stroke population. Caution must be exercised when extrapolating these structural phenotypes to other age intervals. In very elderly patients (e.g., >75 years), pre-existing senile sarcopenia independently alters muscle architecture by adding diffuse muscle thinning and fibro-adipose tissue substitution, which could mask or aggravate stroke-specific phenotypic remodeling. Conversely, younger patients might exhibit higher tissue compliance and distinct neuromuscular adaptation capacities.

Gait asymmetry is mainly based on functional alteration of the paretic side, TA being an essential drive. Researchers also focused on the healthy side and documented overactivity of the non-affected TA, especially in the single-stance phase [8]. Concomitant analysis of both TA muscles, affected and non-affected, could provide more insights into the gait performance.

Structural and electrical alterations in the TA muscle have been documented since the subacute phase of stroke, and they are prone to further evolution [9]. AFO is prescribed early in the subacute phase of stroke rehabilitation and provides adequate support for the ankle and foot, without interfering with TA electrical properties [10].

While clinical assessment and tools like the Modified Ashworth Scale (MAS) are essential for identifying functional hypertonia, they exhibit blind spots regarding muscle structural integrity. MAS cannot differentiate between active neural spasticity and secondary intrinsic tissue remodeling, such as fibro-adipose infiltration or structural contracture. Our study shows that ultrasound imaging transcends these limitations. By quantifying internal metrics like the contraction index and pennation angle, sonography provides an architectural blueprint that prevents clinicians from misinterpreting rigid structural shortening as simple neural spasticity, thereby preventing suboptimal therapeutic or orthotic prescriptions.

This pilot study states that evaluating the tibialis anterior through static metrics or clinical scales alone in chronic stroke survivors is insufficient. Incorporating dynamic ultrasound assessments, i.e., the CI and pennation angle asymmetry, provides an objective framework for post-stroke rehabilitation. Identifying these specific muscular phenotypes should allow the clinician to accurately prescribe orthotics and to optimize therapeutic modalities, such as botulinum toxin injections to reduce spasticity in a rigid phenotype (P1) before orthotic fitting or designing target-specific neuromuscular training for the atrophic phenotype.

### Limitations

The primary limitation of this study is its small sample size (*n* = 8), which characterizes this work strictly as a pilot investigation. Consequently, the documented correlation coefficients did not achieve formal statistical significance (*p* > 0.05) and must be interpreted cautiously as preliminary trends rather than definitive generalized rules. Furthermore, our protocol evaluated the TA muscle in isolation, omitting the multi-muscle assessments across the hip and knee joints required to fully model post-stroke gait dynamics. Future large-scale, longitudinal studies incorporating multi-muscle ultrasound and electromyography are needed to confirm these architectural–functional paradigms.

## 5. Conclusions

In conclusion, this cross-sectional observational pilot study demonstrates that musculoskeletal ultrasound provides critical, objective insights into the structural and architectural remodeling of the tibialis anterior muscle in chronic stroke survivors, revealing essential pathophysiological nuances that static clinical scales cannot capture.

## Figures and Tables

**Table 2:** Complete dataset and contraction index calculation.

	Rest Thickness (cm)	Effort Thickness (cm)	Contraction Index (CI) (Calculated)	Affected Pennation Angle (°)	Unaffected Pennation Angle (°)	Gait Speed (m/s)	MRC Force	Assistive Device
P1	3.560	3.290	0.924	20	12	0.190	1	Cane + AFO
P2	2.815	3.060	1.087	20.50	19.50	0.580	3+	Cane
P3	3.066	3.376	1.1101	1.00	4.66	0.158	3	Cane
P4	2.533	2.683	1.059	10.66	2.33	0.866	4+	AFO
P5	2.950	3.236	1.097	2.66	9.00	0.745	1	AFO
P6	2.296	2.680	1.167	11.66	9.00	0.908	4+	None
P7	2.167	2.535	1.169	4.00	6.00	0.129	1	Cane
P8	2.166	2.620	1.210	7.00	12.33	1.420	1	None

## Data Availability

The raw data supporting the conclusions of this article will be made available by the authors on request.

## References

[1] GBD 2021 Stroke Collaborators (2024). Global, regional, and national burden of stroke and its risk factors, 1990–2021: A systematic analysis for the Global Burden of Disease Study 2021. Lancet Neurol..

[2] Gathia M., Shah N., Kumar S., Singh A. (2023). A comprehensive review of physical therapy interventions for stroke rehabilitation: Impairment-based approaches and goals. Brain Sci..

[3] Juneja P., Hubbard J.B. (2023). Anatomy, Bony Pelvis and Lower Limb: Tibialis Anterior Muscles.

[4] Li S. (2020). Ankle and Foot Spasticity Patterns in Chronic Stroke Survivors with Abnormal Gait. Toxins.

[5] Kim J.H., Chung Y., Kim Y., Hwang S. (2012). Functional electrical stimulation applied to gluteus medius and tibialis anterior corresponding gait cycle for stroke. Gait Posture.

[6] Hwang D.Y., Lee H.J., Lee G.C., Lee S.M. (2015). Treadmill training with tilt sensor functional electrical stimulation for improving balance, gait, and muscle architecture of tibialis anterior of survivors with chronic stroke: A randomized controlled trial. Technol. Health Care.

[7] Cacchio A., Paoloni M., Cimini N., Mangone M., Liris G., Aloisi P., Santilli V., Marrelli A. (2011). Reliability of TMS-related measures of tibialis anterior muscle in patients with chronic stroke and healthy subjects. J. Neurol. Sci..

[8] Tsushima Y., Fujita K., Hayashi K., Kobayashi Y. (2025). Nonparetic tibialis anterior muscle activity during gait: Association with step length asymmetry in chronic stroke patients. Clin. Biomech..

[9] Hu C., Hu H., Mai X., Lo W.L.A., Li L. (2019). Correlation Between Muscle Structures and Electrical Properties of the Tibialis Anterior in Subacute Stroke Survivors: A Pilot Study. Front. Neurosci..

[10] Nikamp C., Buurke J., Schaake L., van der Palen J., Rietman J., Hermens H. (2019). Effect of long-term use of ankle-foot orthoses on tibialis anterior muscle electromyography in patients with sub-acute stroke: A randomized controlled trial. J. Rehabil. Med..

